# Meta-Analysis: Overweight, Obesity, and Parkinson's Disease

**DOI:** 10.1155/2014/203930

**Published:** 2014-02-05

**Authors:** Jinhu Chen, Zhenlong Guan, Liqin Wang, Guangyao Song, Boqing Ma, Yanqin Wang

**Affiliations:** ^1^Department of Endocrinology, Hebei General Hospital, Shijiazhuang 050017, China; ^2^Department of Physiology, College of Life Science, Hebei Normal University, Shijiazhuang 050024, China; ^3^Department of Epidemiology and Statistics, School of Public Health, Hebei Medical University, Shijiazhuang 050000, China

## Abstract

*Objective.* Parkinson's disease (PD) is a severe neurological disease and its risk factors remain largely unknown. A meta-analysis was carried out to investigate the relationship of overweight and obesity with PD. *Methods.* We used PubMed, EMBASE, and the Chinese National Knowledge Infrastructure (CNKI) databases to identify studies of associations between overweight/obesity and PD. Overweight, obesity, and PD were used as keywords, and published works were retrieved until September 30, 2013. The extracted data were classified (BMI ≥ 30, 25 ≤ BMI < 30, and BMI < 25) according to BMI values and analyzed using RevMan5.2 and Stata11.0. *Results.* Four cohort studies and three case-control studies were used to evaluate the association between overweight/obesity and PD, including 2857 PD patients and 5, 683, 939 cases of non-PD controls. There was a statistically significant difference between 25 ≤ BMI < 30  and BMI < 25 in the cohort study (*RR* = 1.17, 95% *CI*, 1.03–1.32,  *P* = 0.03), but there was no difference between BMI ≥ 30  and BMI < 25 or BMI ≥ 30  and 25 ≤ BMI < 30, where the respective *RR* was 1.16 and 0.84; the respective 95% *CI* was 0.67–2.01 and 0.61–1.15, respectively, and the *P* values were 0.60 and 0.28, respectively. Case-control studies showed that there was no statistical difference between any two groups. *Conclusion.* Meta-analysis showed that overweight might be a potential risk factor of PD. Demonstration of a causal role of overweight/obesity in PD development could have important therapeutic implications.

## 1. Introduction

Parkinson's disease (PD) is a severe neurodegenerative disease that results from massive death of dopaminergic neurons in the substantia nigra. However, key pathogenic factors or the pathogenesis of PD remains largely obscure. Previous studies have shown that risk factors such as age, oxidative stress, mitochondrial dysfunction, environmental toxins, nerve inflammation, and genetic factors might be associated with risk of developing PD. In addition, factors that affect an individual's general body shape, such as exercise and diet, also impact on PD. Logroscino had previously shown that a high-fat diet, especially one with increased intake of animal fat, is a risk factor for PD pathogenesis [1]. De Lau et al. and other published works have found that unsaturated fatty acids are factors that are more protective against PD [2].

Globally, there are more than 300 million adults who are obese and 1 billion who are overweight (World Health Organization, WHO, 2010). Obesity and overweight pose a major risk for developing serious chronic diseases [3, 4], including type 2 diabetes [5], cardiovascular disease [6], hypertension and stroke, and Alzheimer's disease [7, 8]. Some studies have suggested that obesity/overweight might be associated with PD [9–12].

Some studies showed that dopamine, a major neurotransmitter in PD, plays important roles in the regulation of food intake [9, 10]. Patients with PD have a loss of dopaminergic neurons and lower dopamine activity in the hypothalamus [11, 12]. Individuals who are suffering from obesity have lower dopamine D_2_ receptor availability in the striatum [13]. Obese people are also usually less active than normal weight people [14, 15], and lower levels of physical activity may increase the risk of developing PD [16]. Although recent epidemiological studies have shown a potential association between obesity/overweight and the risk of PD [17, 18], some studies have not provided evidence in support of this viewpoint [19, 20]. Thus, reported results have been contradictory. Therefore, in this study, we performed a meta-analysis to assess the putative association between obesity/overweight and PD risk.

## 2. Methods 

### 2.1. Search Strategy

We identified all studies that assessed an association between overweight/obesity and the risk of PD in humans. We developed a search engine that was adapted for PubMed, EMBASE, and the China National Knowledge Infrastructure (CNKI) up until September 30, 2013. This was done using the following search terms: “overweight” or “obesity” or “obese” or “adiposity” or “body mass index” (BMI) and “Parkinson disease,” without any language or publication year restrictions. If more than one article was published using the same case series, only the study with the largest sample size was selected. In addition, we examined the reference lists of relevant original papers and review articles.

### 2.2. Selection Criteria and Data Extraction

We included all studies on a case-control or a cohort study of PD, or we examined the association between BMI (BMI ≥ 30, 25 ≤ BMI < 30, and BMI < 25) and PD. We excluded papers without original data, animal data, or* in vitro* studies or other studies that included gene polymorphisms, news stories, commentaries, and letters to the editor.

With the purpose of extracting the necessary characteristics, all relevant articles were collated independently by two reviewers (Jinhu Chen and Yanqin Wang). Both reviewers checked for any encountered discrepancies and reached a consensus. For each selected publication, we focused on the key study characteristics, including publication year, country of origin of where the study was done, the study design, and participant characteristics and demographic information (e.g., gender and body mass index) of both PD patients and control subjects.

### 2.3. Statistical Analysis

All analyses were performed using Review Manager (version 5.1.2) and STATA (version 11.0) software programs. We organized the data, established the database, and verified the collated data according to the requirements of the meta-analysis. Then we calculated the combined effects of the *RR* values or *OR* values and the 95% *CI*, from which we constructed the forest map. We used the *I* ^2^ statistic to investigate the heterogeneity among studies. If there was a statistical difference in terms of heterogeneity (*P* ≤ 0.10), a random-effects model was selected to pool the data. Otherwise, a fixed-effects model was used. Publication bias was evaluated using the funnel plot and the Egger test. Fail-safe numbers were calculated to estimate the stability of the results, *α* = 0.05.

## 3. Results

### 3.1. Study Characteristics

**Figure 1:**
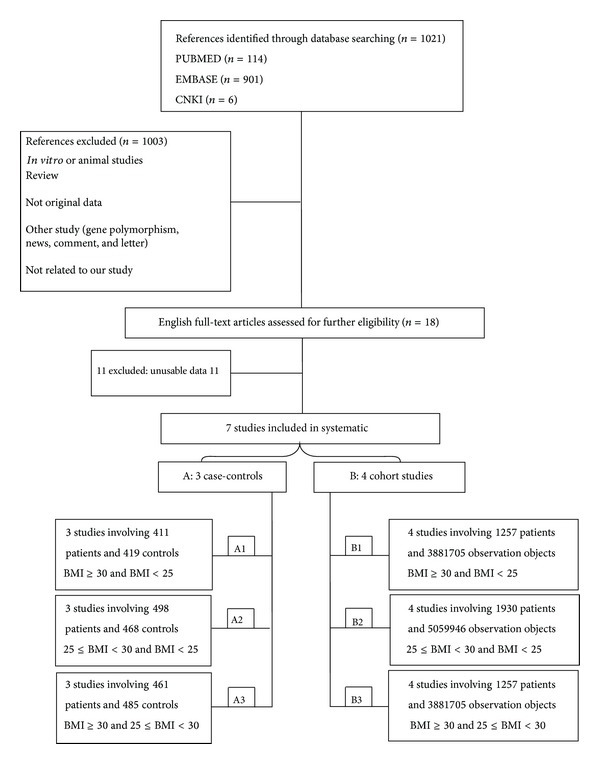
Flow diagram of the study.

A total of seven references that matched the research criteria of the study design were adopted. Among them, three were case-control studies (including 685 PD patients and 686 controls) and four studies were cohort studies (including 2172 PD patients and 5683253 non-PD observation objects). The related literature was published from 2004 to June 2013, including 2857 PD patients and 5683939 non-PD observation objects. The basic characteristics of the study are shown in Table 1. According to the body mass index (BMI), three case-control studies were divided into group A and investigated further. (A1) BMI ≥ 30 versus BMI > 25 (three studies involving 411 patients and 419 controls) [18, 21, 22]; (A2) 25 ≤ BMI < 30 versus BMI > 25 (three studies involving 498 patients and 468 controls) [18, 21, 22]; (A3) BMI ≥ 30 versus 25 ≤ BMI < 30 (three studies involving 461 patients and 485 controls) [18, 21, 22]. According to the BMI, four cohort studies were divided into group B and further analyzed. (B1) BMI ≥ 30 versus BMI > 25 (four studies involving 1257 patients and 3, 881, 705 observation objects) [17, 19, 20, 23]; (B2) 25 ≤ BMI < 30 versus BMI > 25 (four studies involving 1930 patients and 5, 059, 946 observation objects) [17, 19, 20, 23]; (B3) BMI ≥ 30 versus 25 ≤ BMI < 30 (four studies involving 1257 patients and 3, 881, 705 observation objects) [17, 19, 20, 23] (see Figure 1). The results of the meta-analysis are summarized in Table 2.

### 3.2. Qualitative Data Synthesis

**Figure 2:**
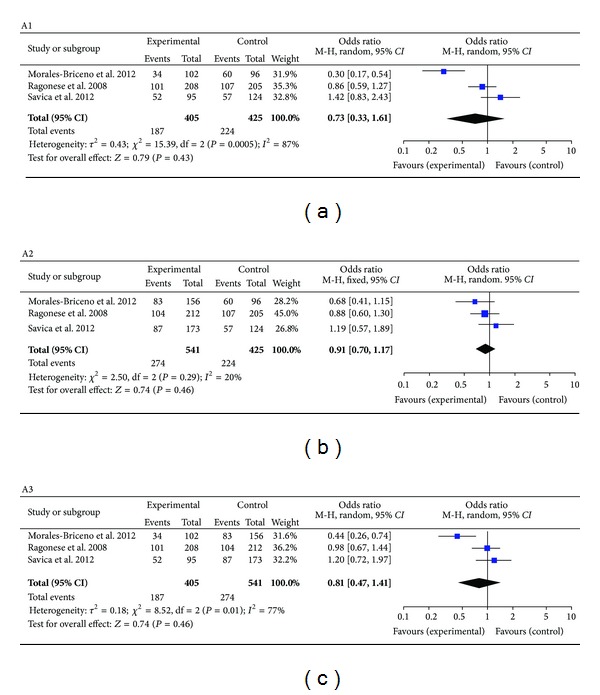
Forest plots for case-control study with all included studies. Meta-analysis of studies reporting BMI and PD versus controls, 95% confidence interval.

**Figure 3:**
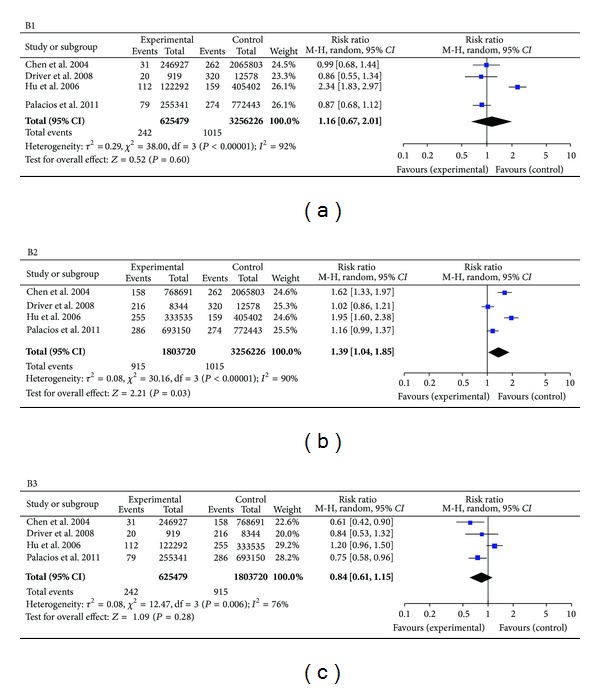
Forest plots for cohort study with all included studies. Meta-analysis of studies reporting BMI and PD versus controls, 95% confidence interval.

Heterogeneities were found within studies of groups A1, A3, B1, B2, and B3. (*I* ^2^ values were 87%, 77%, 92%, 90%, and 76%, resp., and *P* values were =0.0005, <0.01, <0.00001, <0.00001, and =0.006, resp.). A random-effects model was applied for these groups. The combined effects *OR* with a 95% *CI* were, respectively, (A1) 0.73 (0.33, 1.61), (A3) 0.81 (0.47, 1.41), and the combined effects *RR* with a 95% *CI* were, respectively, (B1) 1.16 (0.67, 2.01), (B2) 1.39 (1.04, 1.85), and (B3) 0.84 (0.61, 1.15). There was no heterogeneity observed within studies of groups A2 (*I* ^2^ = 20%, *P* = 0.29). The combined effects *OR* were 0.91 (0.70, 1.17). Overall, there was a statistical difference found between 25 ≤ BMI < 30 and BMI > 25 in the B2 cohort study group. The remainder of the cohort study groups did not display any statistically significant difference (Figures 2 and 3).

Fail-safe numbers for each group, indicating the publication bias, are reported in Table 3. When the meta-analysis results were statistically significant, the minimum number of unpublished studies (the fail-safe number) could be calculated to reverse the conclusion or to bring the meta-analytical mean effect size down to a statistically insignificant level. The greater the fail-safe numbers, the more stable the result. The fail-safe numbers were all relatively large in our meta-analysis, with the exceptions of groups A2 and A3, suggesting that the results were reliable. We further stratified the data according to gender, and the results suggested that no statistical difference was found in the case-control and cohort studies.

### 3.3. Publication Bias

According to the funnel plot and Egger's test, publication bias was not detected for any of the groups.

## 4. Discussion 

This meta-analysis found that 25 ≤ BMI < 30 may increase the risk of Parkinson's disease compared with BMI < 25 in cohort studies, while 25 ≤ BMI < 30 is not a risk factor of Parkinson's disease compared with BMI < 25 in case-control studies. Both case-control studies and cohort studies showed that BMI ≥ 30 did not affect the risk of Parkinson's disease.

This is the first meta-analysis that has examined obesity/overweight and the risk of developing PD, with the novel observation that overweight might be considered as a potential risk factor of developing PD in a cohort study. The proportion of underweight individuals was less when compared with the increasing incidence of overweight and obese individuals. Morales-Briceno et al. [18] reported that overweight and obesity were common among patients with PD, while underweight was almost negligible in Mexico. Barichella et al. [24] found that it was uncommon to find PD patients who were underweight. The main reason for this observation was that overweight people in the Italian population were more prevalent. The classification standard with regard to overweight and obesity differs by nationality and race/ethnicity. In order to maximize information derived from the response data, the standard of overweight and obesity was divided according to BMI criteria as defined by the WHO. Since most studies consolidated underweight and normal weight to BMI < 25, we divided the groups into BMI ≥ 30, 25 ≤ BMI < 30, and a BMI < 25.

We adequately evaluated the role of heterogeneity and publication bias in our study. A random effects model was used in this study, which determined that A1, A3, B1, B2, and B3 were heterogeneous. PD belongs to a class of highly complicated chronic neurodegenerative diseases. The age differences, the duration of the disease, disease severity, weight changes, treatment options, and other factors might all affect the development and prognosis of the disease.

Therefore, the impact of these factors should also be considered. We further analyzed subgroups according to gender based on BMI. The results showed that no statistically significant difference was found between male and female subjects in case-controlled and cohort-based studies. Since most original research literature did not discuss other factors in detail, further subgroup studies were not carried out in this meta-analysis. Further, no obvious publication bias was found by applying Egger's test. In summary, the results of this analysis were reliable.

Bousquet et al. [25] found that exposure to a high-fat diet and consequently overweight/obesity could confer a greater susceptibility to environmental toxins and then accelerate the pathogenesis of PD. Van der Marck et al. [26] considered BMI as an indicator for a meta-analysis and found that PD patients had a significantly lower BMI than controls (overall effect 1.73, 95% *CI*: 1.11–2.35, *P* < 0.001). In this study, we focused on the relationship between overweight/obesity and the risk of PD. The reason for this difference remains unknown, and a potential mechanism might involve obese patients who receive better quality medical treatment, and thus further investigation is warranted.

The present meta-analysis has several limitations. First, the limited number of studies has caused our inability to eliminate the heterogeneity and perform stratified analysis. However, the outcomes of this study demonstrate that we have paid poor attention to this line of investigation. Clearly, it is very important that more research is done in the area of PD, their related conditions, and the incidence and impact of obesity/overweight, particularly with a larger study population size. Second, the fail-safe number also suggested that results from A2 were unstable. However, this data does not affect our conclusions made from the cohort study. Moreover, to eliminate the influence of confounding factors on the results of this study, BMI, gender, and other impacting factors should be considered in multicentered and demographically stratified analyses.

## 5. Conclusions

In summary, cohort studies have shown that overweight could be considered a potential risk for PD. We should strengthen relationship studies aimed at furthering our understanding of overweight, obesity, and PD, pay particular attention to the role that is played by body weight in the onset of PD, and thereby strengthen prevention management strategies in the future.

## Figures and Tables

**Table 1:** Characteristics of the studies included in the meta-analysis.

Author	Date	Country	Type	BMI ≥ 30		25 ≤ BMI < 30		BMI < 25	
				Number of PD	Number of observation objects	Number of PD	Number of observation objects	Number of PD	Number of observation objects
Morales-Briceno et al. [18]	2012	Mexico	Case-control	34	102	83	156	60	96
Ragonese et al. [21]	2008	Italy	Case-control	101	208	104	212	107	205
Savica et al. [22]	2012	USA	Case-control	52	95	87	173	57	124
Hu et al. [23]	2006	Finland	Cohort study	112	122292	255	333535	159	405402
Palacios et al. [19]	2011	USA	Cohort study	79	255341	286	693150	274	772443
Chen et al. [17]	2004	USA	Cohort study	31	246927	158	768691	262	2065803
Driver et al. [20]	2008	USA	Cohort study	20	919	216	8344	320	12578

BMI indicates body mass index (kg/m^2^); PD indicates Parkinson's disease.

**Table 2:** Meta-analysis for all groups.

Group	Number of studies	Reference	Model	Type	*OR*/RR*	95% *CI *		*Z*	*P*
A1	3	[18, 21, 22]	Random-effect	Case-control	0.73	0.33	1.61	0.79	0.43
A2	3	[18, 21, 22]	Fixed-effect	Case-control	0.91	0.70	1.17	0.74	0.46
A3	3	[18, 21, 22]	Random-effect	Case-control	0.81	0.47	1.41	0.74	0.46
B1	4	[17, 19, 20, 23]	Random-effect	Cohort study	1.16*	0.67	2.01	0.52	0.60
B2	4	[17, 19, 20, 23]	Random-effect	Cohort study	1.39*	1.04	1.85	2.21	0.03
B3	4	[17, 19, 20, 23]	Random-effect	Cohort study	0.84*	0.61	1.15	1.09	0.28

A1: BMI ≥ 30 versus BMI < 25; A2: 25 ≤ BMI < 30 versus BMI < 25; A3: BMI ≥ 30 versus 25 ≤ BMI < 30; B1: BMI ≥ 30 versus BMI < 25; B2: 25 ≤ BMI < 30 versus BMI < 25; B3: BMI ≥ 30 versus 25 ≤ BMI < 30. BMI indicates body mass index (kg/m^2^). *indicates *RR* value, without *indicates *OR* value.

**Table 3:** Fail-safe numbers of all groups for studies with no heterogeneity.

Group	Number of studies	Fail-safe number	
		*α* = 0.05	*α* = 0.01
A1	3	10.984	3.928
A2	3	−0.038	−1.533
A3	3	2.748	−0.152
B1	4	25.435	10.583
B2	4	64.381	29.878
B3	4	15.047	5.436

A1: BMI ≥ 30 versus BMI < 25; A2: 25 ≤ BMI < 30 versus BMI < 25; A3: BMI ≥ 30 versus 25 ≤ BMI < 30; B1: BMI ≥ 30 versus BMI < 25; B2: 25 ≤ BMI < 30 versus BMI < 25; B3: BMI ≥ 30 versus 25 ≤ BMI < 30. BMI indicates body mass index (kg/m^2^).
