# Erratum to “Meta-Analysis: Overweight, Obesity, and Parkinson's Disease”

**DOI:** 10.1155/2014/306402

**Published:** 2014-06-25

**Authors:** Jinhu Chen, Zhenlong Guan, Liqin Wang, Guangyao Song, Boqing Ma, Yanqin Wang

**Affiliations:** ^1^Department of Endocrinology, Hebei General Hospital, Shijiazhuang 050017, China; ^2^Department of Physiology, College of Life Science, Hebei Normal University, Shijiazhuang 050024, China; ^3^Department of Epidemiology and Statistics, School of Public Health, Hebei Medical University, Shijiazhuang 050000, China

In the original paper, there were some errors in lines 12, 14, and 19 in the last paragraph on page 2, and in the first line and line 18 on page 3, BMI > 25 should be changed to BMI < 25. There was an unnecessary “a” in the second paragraph of the Discussion section, line 20 on page 3.

